# Effects of Altitude on the Digestion Performance, Serum Antioxidative Characteristics, Rumen Fermentation Parameters, and Rumen Bacteria of Sanhe Heifers

**DOI:** 10.3389/fmicb.2022.875323

**Published:** 2022-04-28

**Authors:** Xinyu Zhang, Shuai Huang, Shengli Li, Wei Wang

**Affiliations:** State Key Laboratory of Animal Nutrition, College of Animal Science and Technology, China Agricultural University, Beijing, China

**Keywords:** high altitude, apparent digestibility, serum antioxidant capacity, rumen microbes, heifers

## Abstract

The production efficiency of dairy cows is affected by altitude, with lower efficiency reported at higher altitudes. However, only a few studies have investigated the digestion performance, serum antioxidative characteristics, rumen fermentation performance, and rumen bacteria of Sanhe heifers at different altitudes. Therefore, in this study, we explored the effects of altitude on these aspects of Sanhe heifers. We evaluated the effects of altitude on the apparent digestibility of nutrients, serum antioxidative characteristics, rumen fermentation parameters, and rumen bacteria in Sanhe heifers. Twenty Sanhe heifers from the same herd and managed with the same practice were used. However, the heifers were from two regions in China: 10 were fed in Hulunbuir City, Inner Mongolia Autonomous Region (119°57′E, 47°17′N; approximately 700 m altitude, named LA) and 10 were fed in Lhasa City, Tibet Autonomous Region (91°06′E, 29°36′N; approximately 3,750 m altitude, named HA). The dry matter intake (DMI), average daily gain (ADG), and DMI/ADG ratio were higher (*p* < 0.05) in LA than in HA heifers, whereas the apparent total tract digestibility of dry matter, ether extract, and crude proteins were higher (*p* < 0.05) in the HA group. Compared with LA heifers, the HA heifers showed decreased (*p* < 0.05) serum concentrations of superoxide dismutase and glutathione peroxidase and increased serum concentration of hydrogen peroxide (*p* < 0.05). Altitude did not significantly affect the volatile fatty acid concentration in the rumen, but HA presented a lower acetate-to-propionate ratio than LA. The 16S rRNA gene sequencing data showed that altitude significantly affected the rumen microbial composition. At the phylum level, the HA heifers presented a lower relative abundance of Actinobacteria (*p* < 0.05) and higher relative abundance of Spirochaetae (*p* < 0.05) than the LA heifers. The correlation analysis revealed that the operational taxonomic units belonging to the genus *Prevotella_1* were correlated (*p* < 0.05) with altitude and DMI. The results indicate that altitude can influence the apparent digestibility of nutrients, serum antioxidant capacity, rumen fermentation, and rumen bacteria composition of Sanhe heifers. The study provides insights into the adaptation mechanism of Sanhe heifers to high-altitude areas.

## Introduction

The Qinghai–Tibetan Plateau is known as the “roof of the world” and “third pole of the earth” (Sun et al., 2021), and it occupies 26% of the Chinese land area (Wu, 2001). Residents of this area rely heavily on animal husbandry to financially support themselves. However, this area is characterized by strong ultraviolet radiation, low atmospheric O_2_ partial pressure, and low temperatures throughout the year (Zha et al., 2016; Sun et al., 2021), which pose several challenges to animals in the region (Cheviron and Brumfield, 2012; Guo et al., 2014).

Qiao et al. (2013a) reported that the production efficiency of dairy cows is lower in high-altitude areas than in low-altitude areas. High-altitude and low-O_2_ environments can cause loss of appetite (Kayser, 1992) and reduced food intake (Gill and Pugh, 1964) in humans and other animals. Some studies have shown that people lose weight after entering a plateau (Lippl et al., 2010). In a high-altitude hypoxic environment, the aerobic metabolism decreases, anaerobic glycolysis levels increase (Cole et al., 2016), and the production capacity of the body might not meet its own needs, which can lead to metabolic disorders, reduced organ function, and microecological imbalance. These effects also occur in other animals and are particularly important to ruminants because their ability to digest feed nutrients depends on the growth and activity of bacteria in their rumen (Deusch et al., 2017; Seshadri et al., 2018). The anaerobic environment in the rumen and the stabilization of the rumen microenvironment are maintained by rumen microorganisms that ferment ingested food into final metabolic products such as volatile fatty acids (VFAs; Mizrahi, 2012). VFAs are absorbed through the rumen wall to meet the energy requirements of animals (Benjamini and Hochberg, 1995). The microbial composition of the rumen is related to animal performance and other production traits (Jewell et al., 2015; Shabat et al., 2016; Zhou et al., 2018); therefore, it can affect the health and production performance of animals. Zhang et al. (2016) reported that the concentration of VFAs produced by rumen fermentation in yaks, which live at high altitudes, was significantly higher than that in cattle, which live at low altitudes; this indicates that altitude affects the digestion and metabolism of rumen microorganisms. However, there are only a few reports on digestion performance and fermentation performance in rumen, and rumen bacteria in dairy cows at different altitudes.

Dairy products such as yogurt, dregs, and butter are a part of the daily diet of Tibetans and an indispensable part of their culture (Beall et al., 1996; Guo et al., 2014). Therefore, milk self-sufficiency in Tibet is highly desired and of importance to locals. Accordingly, the development of the dairy industry in Tibet can potentially improve the dietary structure of Tibetans, enhance their health, and promote an industrial restructure in Tibet. However, high-yielding dairy cows are not adapted to the plateau climate in Tibet; therefore, local milk production does not meet the market demand in the region. To develop the local dairy industry, the Tibet Autonomous Region has introduced Holstein dairy cows (Wang et al., 2018a) and Jersey cattle (Kong et al., 2019) from the inland part of the regions to local areas. A recent study revealed that Holstein dairy cows cannot show long-term adaptability to the plateau environment (Wang et al., 2018a,b). Therefore, the introduction and cultivation of high-quality and high-yielding dairy cow breeds and the development of the dairy cow industry in Tibet should be investigated. Sanhe cattle, which are a dual-purpose breed, are native to the Inner Mongolia Autonomous Region of China. Sanhe cattle are famous for their strong adaptability, disease resistance, feeding tolerance, stable genetic performance, and excellent milk and meat production (Usman et al., 2017; Xu et al., 2017; Hu et al., 2019). Therefore, the growth performance, digestibility, and rumen microbes of Sanhe cattle in high-altitude regions is of interest considering their potential applicability in the Tibet region.

In this study, we investigated the digestion performance of Sanhe heifers in high and low altitudes, and the respective rumen fermentation performance and bacteria. To the best of our knowledge, this is the first study to explore the effects of altitude on digestion performance and rumen fermentation in Sanhe heifers. The results are expected to provide a useful background for the introduction of Sanhe cattle to high-altitude areas.

## Materials and Methods

The protocol was approved by the Ethical Committee of the College of Animal Science and Technology of China Agricultural University (project number AW22121202-1-2).

### Study Regions, Animals, and Their Diet

The two regions investigated in this study were the origin region of Sanhe cattle and the area where they were introduced to, namely Hulunbuir City, Inner Mongolia Autonomous Region (119°57′E, 47°17′N; at ~700 m of altitude, named LA owing to its low altitude), and Lhasa City, Tibet Autonomous Region (91°06′E, 29°36′N; at ~3,750 m, named HA owing to its high altitude), with the average air temperatures of −2.6°C to 1.8°C and 8.57°C, and annual precipitation of 540 and 426.4 mm, respectively.

In June 2020, 200 Sanhe heifers of similar age (born in 2019) and body weight were selected from a herd of 545 Sanhe cattle, which had the same father and were raised in Xieertala farm in Inner Mongolia. For the experiment, 100 Sanhe heifers were maintained in Xieertala farm, whereas the other 100 were sent to Zhizhao dairy farm in Tibet. The experiment was conducted in September 2020. Ten healthy Sanhe heifers were randomly selected from each study region, with a similar age of 14–15 months and mean body weights of 340.71 and 329.80 kg, and the experiments were conducted for 30 days. Before sampling, Sanhe heifers were maintained under the same feedlot conditions for 2 weeks and were fed *ad libitum*, during which they were fed a total mixed ration (TMR) of similar nutrient levels (dry matter (DM) basis; Supplementary Table S1) twice daily (06:30 and 08:30, 17:30 and 19:30 h). The TMR was formulated to ensure that the nutrient requirements for dairy heifers were met or exceeded under the given conditions. Water was freely available per the guidelines of the National Research Council (NRC, 2001). Sanhe heifers were housed in individual stalls in a ventilated, environmentally controlled tie-stall barn with rubber mattress bedding. During the experiment, each heifer was weighed daily for feed intake and residual. At 16 and 30 days, the heifers were weighed at 06:00 and 08:00 h, before morning feeding, to adjust the amount of feed offered.

### Dry Matter Intake and Digestibility Measurements

Periods of 2 and 1 week were adopted to allow heifers to adapt to their diet and individual stalls, respectively. In the last week of the experiment, the dry matter intake (DMI) of each heifer was recorded daily. Feed and fecal samples were analyzed for internal tract digestibility. TMR samples and individual feedstuffs were collected and dried daily for analysis. The total feces were collected over the last 3 days of the experiment by manure collection according to the method of Cao et al. (2009). We weighed the feces and cleaned the stalls each day of the last 3 days. Moreover, 4% of all fresh fecal samples were collected daily from each Sanhe heifer for 3 consecutive days. Tartaric acid (10%) was added to the samples and mixed well before drying for 48 h at 65°C in an air-forced oven. All feed and fecal samples were ground and passed through a 1-mm sieve, and their DM, ether extract (EE), ash, and crude protein (CP) content were measured according to the methods of AOAC (Lee, 1995). The content of neutral detergent fiber (NDF) and acid detergent fiber (ADF) was determined according to the method of Van Soest et al. (1991). The organic matter (OM) content was equal to the DM subtracted by its ash content.

### Blood Collection and Analysis

On the last day of the trial, blood samples were collected from each heifer *via* the tail vein before the morning feeding. Samples were centrifuged at 3,000 × *g* for 10 min to obtain the serum, which was stored at −20°C until analysis. The serum samples were analyzed for total antioxidant capacity (T-AOC) and superoxide dismutase (SOD), catalase (CAT), malondialdehyde (MDA), glutathione peroxidase (GSH-Px), hydrogen peroxide (H_2_O_2_), and glutathione (GSH) levels, which were determined using spectrophotometry (Leng GuangSFZ1606017568; Shanghai, China), with a colorimetric kit according to the manufacturer’s instructions (Nanjing Jiancheng, Jiangsu, China).

### Rumen Sample Collection and Processing

At the end of the experiment, 20 ruminal chyme samples (one from each heifer) were collected at 7:00 h, before morning feeding, using an oral gastric tube (Ancitech, Winnipeg, MB, Canada). The sampling device was cleaned with fresh water before each sampling, and the first 200 ml of rumen sample was discarded. Subsequently, 100 ml of the collected ruminal chyme was filtered using four layers of cheesecloth to obtain the rumen fluid, which was immediately frozen in liquid nitrogen (−196°C) for the analysis of rumen bacteria and stored at −20°C for the examination of rumen fermentation parameters. One samples was used for bacteria analysis and another for rumen fermentation analysis.

To analyze the VFA concentrations (including acetate, propionate, butyrate, and valerate), the rumen fluid of each specimen was thawed and centrifuged at 8,000 × *g* for 15 min at 4°C to obtain the supernatant, which was then quantified using gas chromatography as described by Erwin et al. (1961).

### Genomic DNA Extraction, PCR Amplification, and 16S rRNA Sequencing

The total microbial genomic DNA was extracted from 1-ml samples of rumen fluid using the OMEGA kit (Omega Bio-Tek, Norcross, GA, United States) according to the manufacturer’s instructions. The concentration and purity of the extracted DNA were confirmed using a Nanodrop 2000 spectrophotometer (Thermo Fisher Scientific, Waltham, MA, United States). The V3–V4 region of the rumen bacterial 16S rRNA gene was amplified using the forward primer *338F* (5′-ACTCCTACGGGAGGCAGCA-3′) and reverse primer *806R* (3′-GGACTACNNGGGTATCTAAT-5′). The PCR conditions were as follows: initial denaturation at 95°C for 5 min, followed by 28 cycles at 95°C for 45 s, 55°C for 50 s, 72°C for 45 s, and a final extension at 72°C for 10 min. The amplified fragments were subjected to 2% agarose gel electrophoresis to detect amplicons, purified using the Agencourt AMPure XP kit (Beckman Coulter Genomics, Indianapolis, IN, United States) according to the manufacturer’s instructions, and then quantified using QuantiFluor-ST (Promega, United States). Following standard protocols, the purified PCR products were sequenced on Illumina MiSeq (Illumina, San Diego, CA, United States; Caporaso et al., 2012), with a 2 × 250 bp sequencing kit.

### Quality Control and Statistical Analysis

QIIME 1.8 (Caporaso et al., 2010) was used to filter and remove sequences with scores ≤20 (low quality), reads <200 bp, and reads containing ambiguous bases or unmatched to primer sequences, and the respective barcode tags were removed. The remaining sequences were combined *via* PEAR 0.9.6 (Zhang et al., 2014) and demultiplexed using Flash (version 1.20; Magoč and Salzberg, 2011). Reads with a combined length of <230 bp and chimeric sequences were removed using the UCHIME algorithm (Edgar et al., 2011). To reduce the errors attributed to the different sequencing depths, all samples were subsampled to an equal size of 32,813 sequences for a downstream alpha and beta diversity analysis. To ensure the comparability of species diversity between the samples, standardized operational taxonomic unit (OTU) documents were used to analyze the species and diversity indices.

The resulting sequences were then clustered into OTUs based on a 97% sequence similarity threshold using the Ribosomal Database Project classifier (Cole et al., 2009) with a confidence threshold of 0.70 and compared against the SILVA 128 database for microbial species annotation (Quast et al., 2012). All values were obtained using UCLUST to generate a representative OTU table (Edgar, 2010).

The OTU-level alpha diversity of bacterial communities was determined using procedures in QIIME 1.8 and the Chao1, Shannon, and Simpson indices; the results were visualized using the “ggplot2” package of R (version 4.0.5; Ginestet, 2011). The principal coordinates analysis (PCoA) based on Bray–Curtis dissimilarity matrices was conducted in R using the “vegan” package for beta diversity analysis (Oksanen et al., 2016). PICRUSt 2 was used to predict the functional differences in the rumen bacteria of the samples from the two regions.^1^

### Statistical Analysis

Data of DMI, nutrient digestibility, serum biochemistry indices, and rumen fermentation were analyzed using the *t*-test in SPSS (version 22.0, IBM SPSS, Chicago, Illinois, United States). Alpha diversity indices, which represented the significance of the pairwise comparison between the LA and HA groups, were analyzed using Wilcoxon rank test with the “dplyr” package version, 0.7.6 (Wickham, 2017) in R. The PCoA was performed in R based on the Bray–Curtis dissimilarity matrices, and the “ggplot2” package (Ginestet, 2011) in R was used for visualization. The differences in the relative abundances at the phylum, family, and genus levels and the bacteria functions of the two groups were investigated using Wilcoxon test in R (version 4.0.5). Spearman’s rank correlation was used to identify the correlations among the relative abundance of core OTUs, DMI, altitude, rumen fermentation parameters, and serum antioxidant indices using the “Psych” package version, 1.6.9 (Revelle, 2018), and the results were visualized using the “corrplot” package version, 0.84 (Wei and Wei, 2016) in R. All data are reported as mean, and significance was established at *p* < 0.05.

## Results

### Growth Performance and Apparent Digestibility of Nutrients in Sanhe Heifers

The effects of altitude on the growth performance and apparent digestibility of nutrients in Sanhe heifers are shown in Table 1. The DMI and ADG were significantly higher (*p* < 0.05) in LA heifers than in HA heifers, whereas the DMI/ADG and apparent total tract digestibility of DM, CP, and EE were significantly higher (*p* < 0.05) in HA heifers than in LA heifers. There were no significant differences (*p* > 0.05) in the apparent total tract digestibility of OM, NDF, and ADF between the groups.

**Table 1:** Effect of altitude on the growth and apparent digestibility of nutrients in Sanhe heifers.

Parameter	Group^1^		SEM	*p*
	LA	HA		
**Growth performance**				
Initial body weight, kg	340.71	329.80	3.99	0.25
15 Days final body weight, kg	350.15	337.40	3.92	0.21
Average daily gain (ADG, kg)	0.63	0.50	0.03	0.01
Dry matter intake (DMI, kg/d)	10.38	8.53	0.32	<0.01
DMI/ADG (kg/kg)	15.55	17.32	0.36	<0.001
**Apparent total tract digestibility of nutrients (%)**				
Dry matter (DM)	58.31	62.77	0.09	0.03
Crude protein (CP)	71.24	73.02	1.04	0.03
Organic matter (OM)	60.69	68.09	1.88	0.11
Ether extract (EE)	77.85	83.80	1.10	0.02
Neutral detergent fiber (NDF)	41.47	43.98	1.06	0.91
Acid detergent fiber (ADF)	40.22	40.08	1.21	0.52

1 Low-altitude (LA) and high-altitude (HA) groups.

### Serum Antioxidant Enzyme Activity and Oxidant Products

The levels of SOD and GSH-Px in the serum were lower (*p* < 0.05) in the HA group than in the LA group. The HA heifers also showed higher (*p* < 0.05) H_2_O_2_ concentrations in the serum than LA heifers. However, there were no significant differences in the content of T-AOC, GSH, MDA, and CAT between the groups (Table 2).

**Table 2:** Effect of altitude on the serum antioxidant enzyme activities and oxidant products in Sanhe heifers.

Items	Groups^1^		SEM	*p*
	LA	HA		
Total antioxidant capacity (T-AOC, U/ml)	9.78	10.73	0.31	0.13
Superoxide dismutase (SOD, U/ml)	51.80	50.25	0.35	0.02
Glutathione peroxidase (GSH-Px, U/ml)	6.61	4.19	0.62	0.04
Reduced glutathione (GSH, μg/ml)	29.68	28.81	2.49	0.87
Malondialdehyde (MDA, nmol/ml)	1.05	1.24	0.06	0.10
Catalase (CAT, U/ml)	9.42	9.43	0.21	0.97
Hydrogen peroxide (H_2_O_2_, mmol/L)	54.17	65.91	2.38	0.01

1 Low-altitude (LA) and high-altitude (HA) groups.

### Rumen Fermentation Parameters

There was no significant difference in acetate, propionate, butyrate, valerate, and total volatile fatty acid (TVFA) concentrations (*p* > 0.05) between the groups. Furthermore, a higher acetate-to-propionate (AP) ratio was observed in LA heifers than in HA heifers (*p* < 0.05; Table 3).

**Table 3:** Parameters of rumen fluid fermentation in Sanhe heifers at different altitudes.

Parameter	Group^1^		SEM	*p*
	LA	HA		
pH	6.70	6.74	0.06	0.45
Acetate (mmol/L)	77.08	72.26	3.62	0.52
Propionate (mmol/L)	23.24	23.08	1.18	0.95
Butyrate (mmol/L)	13.78	12.12	0.88	0.36
Valerate (mmol/L)	2.06	1.98	0.16	0.72
^2^Acetate/propionate (AP)	6.72	6.28	0.08	<0.05
^3^TVFAs (mmol/L)	117.52	114.22	5.80	0.71

1 Low-altitude (LA) and high-altitude (HA) groups.

2 AP, acetate-to-propionate ratio.

3 TVFA, total volatile fatty acid.

### Ruminal Bacteria in Sanhe Heifers at Different Altitudes

#### Sequencing Metrics for the Rumen Bacteria of Heifers

**Figure 1:**
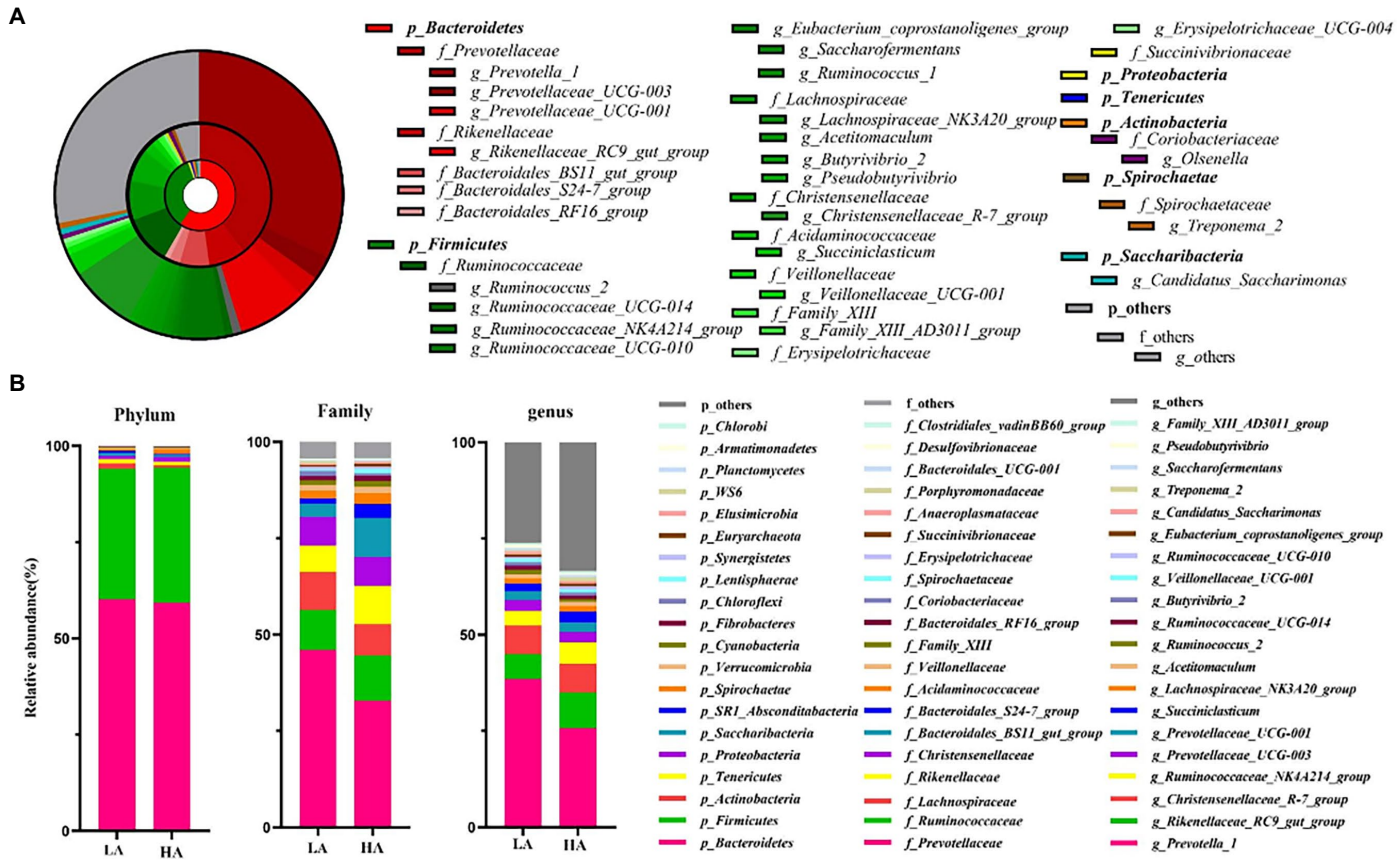
Composition of ruminal bacteria in Sanhe heifers. **(A)** Relative distribution of the most dominant bacterial phyla, families, and genera (relative abundance >0.5% for 20 samples) in the LA and HA groups. The inner, middle, and outer rings represent the phylum, family, and genus levels, respectively. Different shades of color represent different bacteria. **(B)** Top 20 average relative abundance of phyla, family, and genus in the LA and HA groups. LA, low-altitude group and HA, high-altitude group.

A total of 867,544 raw sequences were generated, with an average of 43,377 ± 882 (mean ± SD) per sample. An average of 2,497 ± 53 OTUs were identified across all samples, with 97% sequence similarity. Rarefaction curves showed a smaller number of newly identified OTUs because the number of sequences per sample increased (Supplementary Figure S1). This indicated an adequate sampling depth for the analysis of rumen bacterial composition. A good sequence coverage was achieved, with a mean value of 0.968 across all 20 samples. The three most dominant abundant phyla were Bacteroidetes (59.72%), Firmicutes (34.45%), and Actinobacteria (1.02%; Figures 1A,B). Within these phyla, Prevotellaceae (39.49%), Ruminococcaceae (11.01%), Lachnospiraceae (9.02%), Rikenellaceae (8.36%), Christensenellaceae (7.48%), and Bacteroidales_BS11_gut_group (6.75%) were the most abundant families (Figures 1A,B). At the genus level, eight genera presented relative abundance of >2%, namely *Prevotella_1* (32.15%), *Rikenellaceae_RC9_gut_group* (7.86%), *Christensenellaceae_R-7_group* (7.44%), *Ruminococcaceae_NK4A214_group* (4.66%), *Prevotellaceae_UCG-003* (2.73%), *Prevotellaceae_UCG-001* (2.44%), *Succiniclasticum* (3.97%), and *Romboutsia* (2.41%; Figures 1A,B).

#### Core Bacteria in Sanhe Heifers

**Figure 2:**
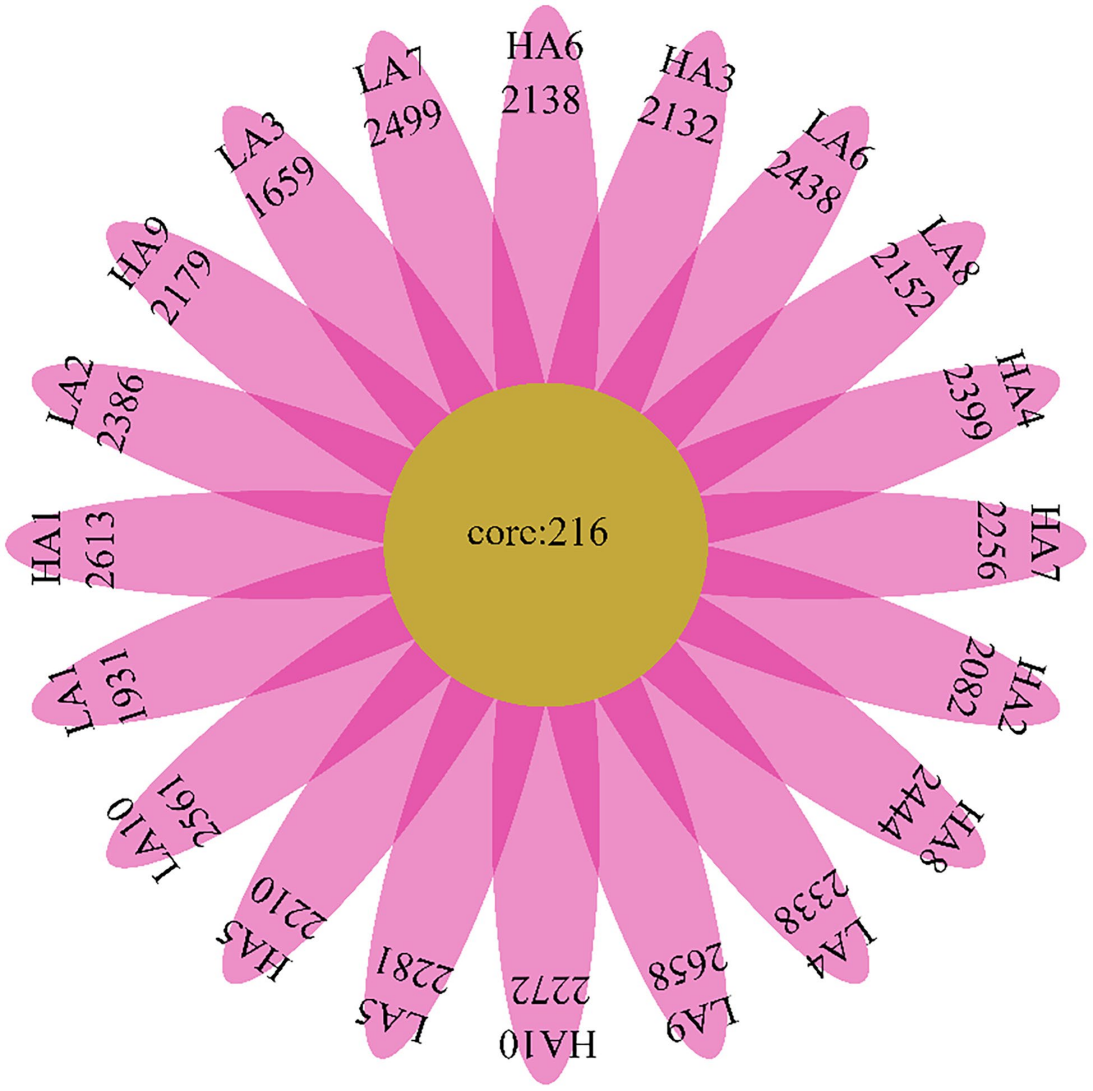
Flower diagram plot of Sanhe heifer samples. The core community of all Sanhe heifers was defined as the operational taxonomic units present in all Sanhe heifers throughout the sampling period.

We determined the core bacteria in all Sanhe heifer specimens analyzed in this study and identified 216 OTUs shared among all samples (Figure 2). These included bacterial families with total relative abundance of >1%, including Prevotellaceae (28.67%), Lachnospiraceae (2.52%), Veillonellaceae (1.78%), Rikenellaceae (1.72%), Acidaminococcaceae (1.60%), and Ruminococcaceae (1.58%; Supplementary Table S1). The shared genera among all samples with total relative abundance of >1% were *Prevotella_1* (12.87%), *Christensenellaceae_R-7_group* (4.84%), *Ruminococcaceae_NK4A214_group* (3.98%), *Rikenellaceae_RC9_gut_group* (3.89%), *Succiniclasticum* (2.13%), and *Prevotellaceae_UCG-003* (1.04%; Supplementary Table S1).

#### Ruminal Bacterial Community of LA and HA Sanhe Heifers

**Figure 3:**
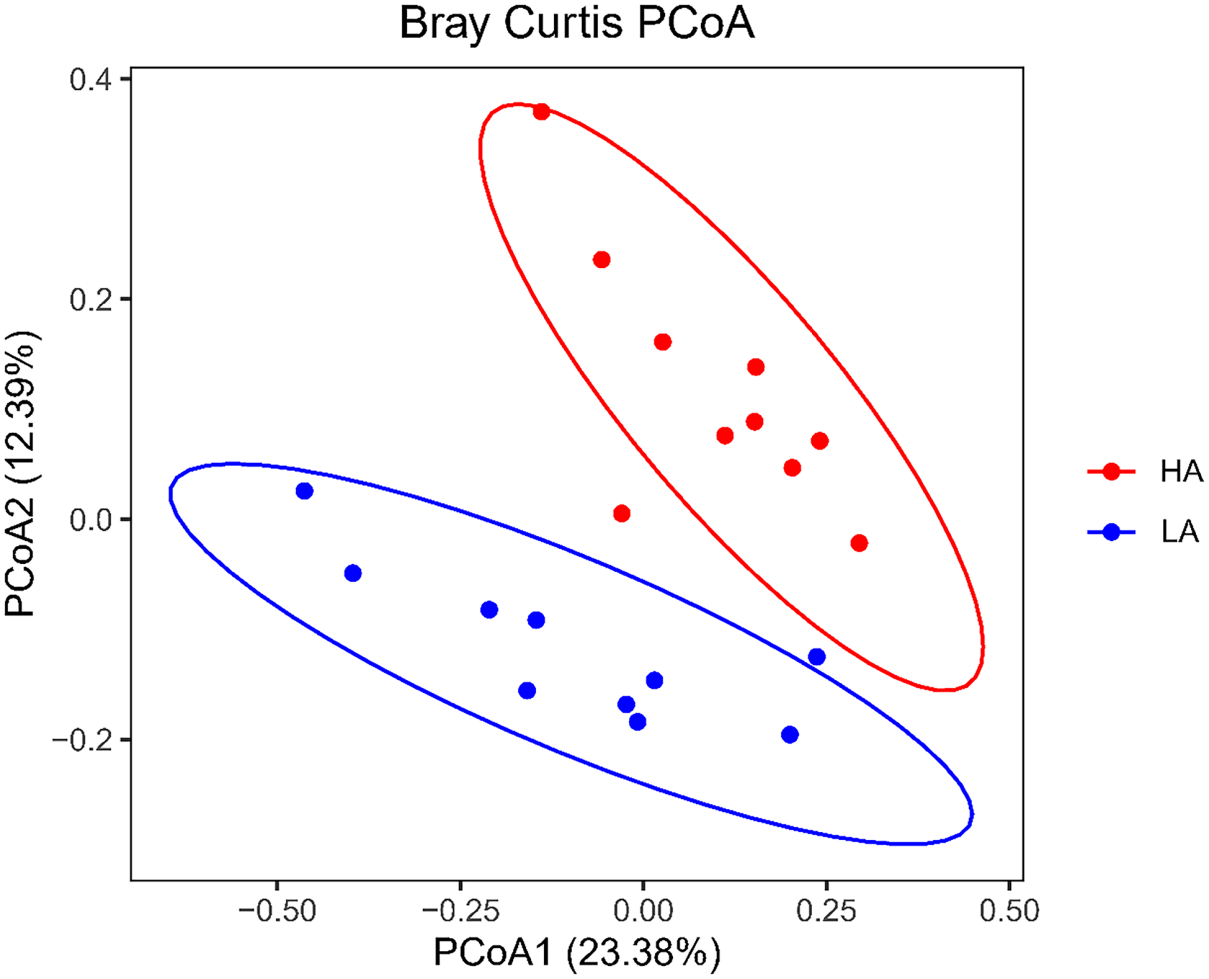
Principal coordinates analysis (PCoA) plots of bacterial communities in Sanhe heifer rumen samples. LA, low-altitude group and HA, high-altitude group.

There were no differences (*p* > 0.05) in the rumen bacteria between LA and HA Sanhe heifers in terms of Chao1 richness and Shannon diversity index values (Supplementary Figure S2). We performed Bray–Curtis dissimilarity analysis and visualized the results using a PCoA plot to identify possible differences between the ruminal bacteria of LA and HA Sanhe heifers, as shown in Figure 3. We analyzed these data in detail using ANOSIM and confirmed that these two groups were statistically different (*R*^2^ = 0.362, *p* = 0.001). At the phylum level, the relative abundance of Bacteroidetes, Firmicutes, Tenericutes, Proteobacteria, and Fibrobacteres in the LA and HA groups showed no significant differences (*p* > 0.05). The HA group presented lower (*p* < 0.05) relative abundance of the phylum Actinobacteria, and higher (*p* < 0.05) relative abundance of the phylum Spirochaetae. At the family level, Bacteroidales_BS11_gut_group, Bacteroidales_S24-7_group, and Spirochaetaceae were significantly more predominant (*p* < 0.05) in the HA group than in the LA group (Table 4). The relative abundance of Coriobacteriaceae was significantly lower (*p* < 0.05) in HA than in LA heifers (Table 4). At the genus level, the relative abundance of 18 genera was significantly higher (*p* < 0.05) and that of 11 genera was significantly lower in the HA group than in the LA group (Supplementary Table S2). Moreover, the relative abundance of some genera changed by more than 10-fold in the LA group compared with that in the HA group, including *Lachnospira* (15.72-fold decrease, *p* < 0.001), *Corynebacterium_1* (18.50-fold decrease, *p* < 0.001), *Howardella* (14.22-fold decrease, *p* < 0.001), *Solanum torvum* (39.01-fold increase, *p* < 0.001), *Lactobacillus* (13.99-fold increase, *p* < 0.001), and *Pyramidobacter* (27.50-fold increase, *p* < 0.001).

**Table 4:** Phyla and families with relative abundance of >0.1% in the rumen bacteria of LA and HA.

Phylum/Family	Group^1^		SEM	*p*
	LA	HA		
**Bacteroidetes**	**60.18**	**59.27**	**0.030**	**0.971**
Prevotellaceae	46.06	32.92	4.325	0.218
Rikenellaceae	6.84	9.89	0.738	0.089
Bacteroidales_BS11_gut_group	3.38	10.13	0.933	<0.001
Bacteroidales_S24-7_group	1.37	3.63	0.615	0.011
Bacteroidales_RF16_group	1.11	1.31	0.165	0.315
Porphyromonadaceae	0.62	0.19	0.192	0.272
Bacteroidales_UCG-001	0.24	0.39	0.043	0.075
**Firmicutes**	**33.86**	**35.05**	**0.031**	**0.912**
Ruminococcaceae	10.34	11.69	1.018	0.529
Lachnospiraceae	9.89	8.15	0.803	0.353
Christensenellaceae	7.46	7.49	1.020	0.796
Acidaminococcaceae	1.99	2.83	0.429	0.631
Veillonellaceae	1.52	1.6	0.327	1.000
Family_XIII	1.19	1.6	0.237	0.352
Erysipelotrichaceae	0.77	0.6	0.159	0.850
Clostridiales_vadinBB60_group	0.16	0.21	0.032	0.089
**Actinobacteria**	**1.42**	**0.62**	**0.002**	**0.015**
Coriobacteriaceae	1.27	0.61	0.139	0.014
**Tenericutes**	**1.11**	**0.97**	**0.001**	**0.684**
Anaeroplasmataceae	0.5	0.46	0.061	0.909
**Proteobacteria**	**0.87**	**1.21**	**0.003**	**0.791**
Succinivibrionaceae	0.4	0.84	0.338	0.880
Desulfovibrionaceae	0.25	0.19	0.034	0.677
**Spirochaetae**	**0.41**	**1.14**	**0.002**	**0.011**
Spirochaetaceae	0.41	1.14	0.155	0.009
**Fibrobacteres**	**0.13**	**0.14**	**0.002**	**0.880**
Fibrobacteraceae	0.13	0.14	0.023	0.880

1 Low-altitude (LA) and high-altitude (HA) groups. The bold values is the bacteria at phylum.

#### Correlation of Ruminal Bacteria With Altitude, DMI, and Rumen Fermentative Parameters

**Figure 4:**
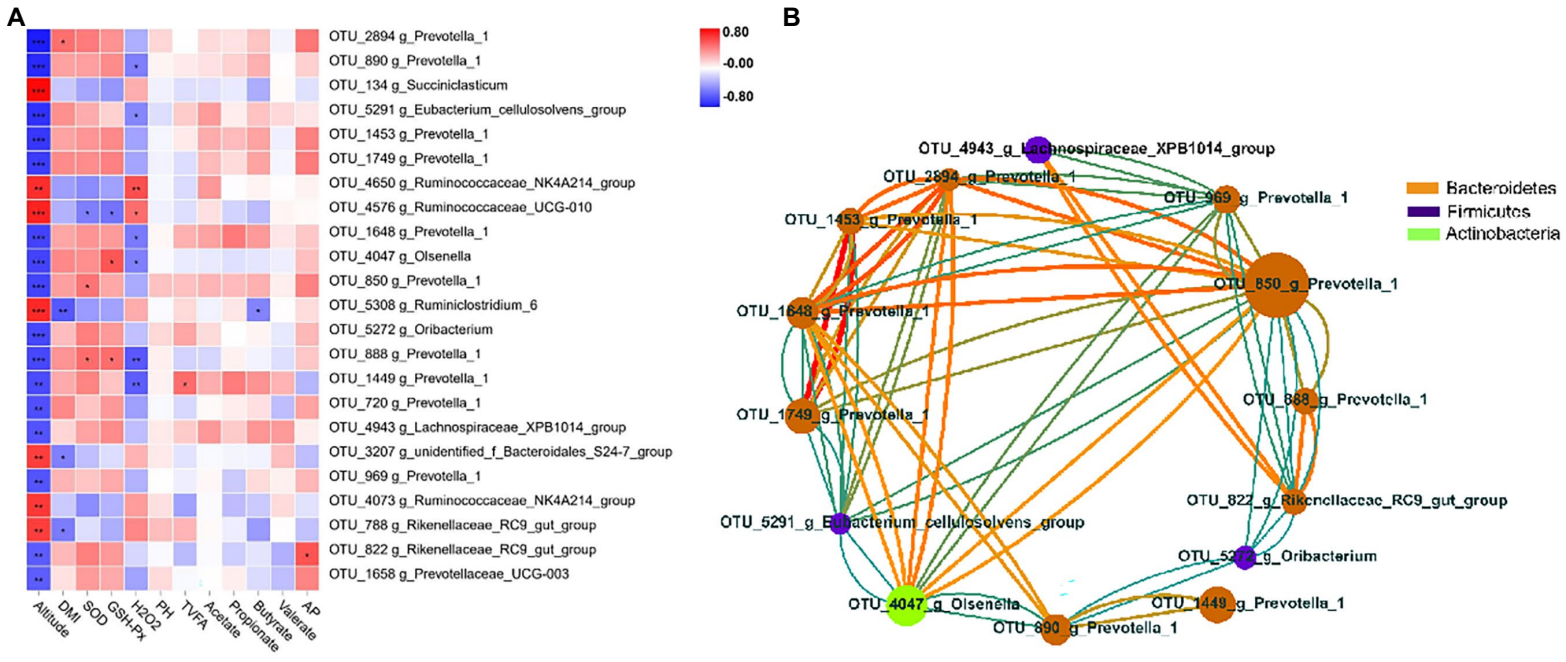
Correlation analysis of rumen bacteria. **(A)** Heatmap of core operational taxonomic units (OTUs) (relative abundance >0.01% and *p* < 0.05 in all samples) significantly associated with production and fermentative parameters of feces in low- and high-altitude Sanhe heifers, as determined using the Spearman’s correlation analysis. ^*^0.01 < *p* < 0.05; ^**^0.001 < *p* < 0.01; and ^***^p ≤ 0.001. **(B)** Interaction among relative abundance >0.01% and *p* < 0.05 of OTUs (red = positive correlation; blue = negative correlation; size and color of circles = relative abundance of OTUs and the genus to which they belong, respectively).

To explore the potential function of ruminal bacteria on production and fermentation, we analyzed the correlation among altitude, VFAs (acetate, propionate, butyrate, valerate, total VFAs, and AP), and the relative abundance of OTUs using Spearman’s rank correlations. OTUs with relative abundance of <0.01% in all samples were excluded from further analysis, and we selected OTUs with significant differences in the remaining OTUs. The correlations of OTUs with altitude and fermentation traits are shown in Figure 4A. The results showed that 23 OTUs were significantly correlated (*p* < 0.05) with altitude. Of these 23 OTUs, 16 were negatively correlated with altitude, 10 of which were of the genus *Prevotella_1* (*p* < 0.05), and 11 were of the family Prevotellaceae (*p* < 0.05). Seven OTUs were positively correlated with altitude, four of which belonged to the family Ruminococcaceae. Moreover, a significant negative correlation (*p* < 0.05) with DMI was observed for three genera, namely, *Rikenellaceae_RC9_gut_group*, *Ruminiclostridium_6*, and *g_unidentified_f_Bacteroidales_S24-7_group*. One OTU belonging to the genus *Prevotella_1* was significantly positively correlated (*p* < 0.05) with DMI. For VFAs, the TVFA concentration was positively correlated (*p* < 0.05) with one OTU within the genus *Prevotella_1*, whereas the butyrate concentration was negatively correlated (*p* < 0.05) with one OTU within the genus *Ruminiclostridium_6*. One OTU belonging to the genus *Rikenellaceae_RC9_gut_group* was positively correlated (*p* < 0.05) with the AP ratio. Two OTUs belonging to the genus *Prevotella_1* were significantly positively associated (*p* < 0.05) with SOD, whereas one OTU belonging to the genus *Ruminococcaceae_UCG-010* was significantly negatively associated (*p* < 0.05) with it. Two OTUs belonging to the genera *Prevotella_1* and *Olsenella* were significantly positively associated (*p* < 0.05) with GSH-Px, whereas one OTU belonging to the genus *Ruminococcaceae_UCG-010* was significantly negatively associated (*p* < 0.05) with it. Two OTUs belonging to the genera *Ruminococcaceae_UCG-010* and *Ruminococcaceae_NK4A214_group* were significantly positively associated with H_2_O_2_, whereas six OTUs belonging to the genera *Prevotella_1*, *Olsenella*, and *Eubacterium_cellulosolvens_group* were significantly negatively associated with it. To better clarify the interactions of core OTUs (relative abundance >0.01%, *p* < 0.05) for the two groups, a co-occurrence network was developed. The OTUs belonging to the phyla Bacteroidetes, Firmicutes, and Actinobacteria presented a strong interaction correlation (|*r*| > 0.6, *p* < 0.05; Figure 4B).

### PICRUSt2 Function Prediction

The function of rumen microbial communities in Sanhe heifers was predicted using PICRUSt2 software, and the differences in KEGG pathway enrichment between the LA and HA groups were determined (Supplementary Table S3). The results of Wilcoxon test showed that 17 pathways presented significant differences between the groups (*p* < 0.05; Supplementary Table S3). The pathway with the highest relative abundance in both groups was the biosynthesis of the vancomycin group of antibiotics (2.51%), followed by thiamine metabolism (1.69%). PICRUSt2 software enriched 45 dominant pathways (relative abundance >1%) in level III KEGG pathways.

## Discussion

The DMI and ADG of Sanhe heifers decreased with altitude because when animals enter high-altitude areas, the sympathetic nerve activation increases and releases stress hormones that increase the body’s metabolism (Chen et al., 2007), thereby suppressing appetite and reducing DMI, which eventually decreases ADG. Previous studies (Gill and Pugh, 1964; Kayser, 1992; Lippl et al., 2012) have shown that high altitudes can decrease human appetite and cause weight loss, which is consistent with the results of this study. However, Qiao et al. (2013a) reported that a high altitude did not affect the DMI of Chinese Holstein cows, which is contrary to our results. This might be attributed to the differences in cattle breed, study region, and environment. The apparent total tract digestibility of CP and EE was higher in the HA group than in the LA group likely because of the insufficient energy intake owing to the decreased DMI, which leads the body to compensate by increasing the digestibility of CP and EE to meet energy requirements (Wang et al., 2020). Therefore, the increase in the apparent total tract digestibility of CP and EE of Sanhe heifers at high altitudes might occur as a compensation to the decrease in DMI and digestibility of DM. Therefore, the results suggest that 3 months after entering Tibet, Sanhe heifers adjusted their apparent digestibility of nutrients to meet their nutritional needs. However, Qiao et al. (2013b) used *in vitro* culture to measure the effects of different altitudes on Holstein dairy cows and reported no significant changes in the degradation of nutrients, which is not consistent with our results. Possible reasons for this are the differences in cattle breed, which can lead to different adaptabilities to plateau environments; altitudes, as Qiao et al. (2013b) investigated areas at 1,600 and 3,600 m (lower altitude difference than that in our study); and experimental conditions, as the current study is an animal digestion experiment, whereas the previous study was an *in vitro* analysis.

Exposure to highland hypoxia can cause biochemical and metabolic changes because of lower availability of O_2_ and decreased activity and content of antioxidant enzymes (Frisancho, 2013). Hypoxia can lead to the formation of reactive oxygen species by auto-oxidation of one or more mitochondrial complexes (Chandel et al., 1998), which leads to oxidative stress (Maiti et al., 2006) and further oxidative damage (Zhao et al., 2009). Antioxidant enzymes, including SOD, GSH-Px, and CAT, are cellular defenses (Radák et al., 1994; Nagaraja and Titgemeyer, 2007). In the oxidative stress response system, H_2_O_2_ is a chemically active reactive oxygen species, whereas GSH-Px is synthesized in the kidney and secreted into the blood as an important antioxidant enzyme, which can scavenge reactive oxygen species in extracellular compartments and remove the produced H_2_O_2_ during normal metabolism or after oxidative damage (Bierl et al., 2004; Jin et al., 2011). Xu et al. (2014) reported that low O_2_ exposure leads to low SOD activity, which is consistent with our results. In our experiment, the GSH-Px level in the serum was lower in the HA group than in the LA group, whereas the H_2_O_2_ level was higher in the HA group. Therefore, we suggest that the balance of oxygen free radicals in the cells was disturbed by the high-altitude hypoxic environment, and the antioxidant capacity of Sanhe cattle decreased with increasing altitude.

Understanding the factors that influence the composition and function of the ruminal bacteria can benefit the maintenance of homeostasis in the digestive tract of ruminants. Therefore, one of the objectives of this study was to characterize the ruminal bacteria in Sanhe heifers at LA and HA and determine the correlation among altitude, DMI, VFAs, and ruminal bacteria. Understanding the dynamics of the ruminal bacteria of Sanhe heifers can provide a framework for feed management.

In this study, there were no changes in the VFAs in ruminal samples between the regions, suggesting that the rumen fermentation performance of Sanhe heifers had little effect. Moreover, Bacteroidetes and Firmicutes occupied the dominant positions in both groups. Bacteroidetes members are the most important bacteria in the rumen from birth to adulthood, and Firmicutes members are crucial for energy conversion. They are important for the digestion of proteins and carbohydrates, respectively (Liu W. et al., 2021). From the above results, the beta diversity between the LA and HA groups was also significantly different. Therefore, the results indicate that altitude can significantly affect the bacterial structure and composition in the rumen of Sanhe heifers.

*Lachnospira*, belonging to the family Lachnospiraceae, is one of the main VFA producers and is closely related to other cellulose-degrading bacteria (Myer et al., 2015). Shabat et al. (2016) suggested that Lachnospiraceae is associated with the feed efficiency of dairy cows. *Corynebacterium_1* belongs to the phylum Actinobacteria, and it is generally facultatively anaerobic and fermentative and metabolizes lactate (Gardell, 1980). *Pyramidobacter* can anaerobically degrade fibers and produce VFAs (Kang et al., 2020). In addition, the relative abundance of *Lachnospira*, *Howardella*, and *Lactobacillus*, which belong to the phylum Firmicutes, changes with altitude, mostly because of the differences in fiber fermentation and cellulose degradation. A previous study performed high-throughput sequencing of 742 ruminant rumen samples from 35 countries and reported that geographic distribution also affected the rumen microbial community (Henderson et al., 2015), and this is consistent with our results. This phenomenon was attributed to the thermoregulatory challenges and hypoxic stress experienced by animals at high altitudes (Liu K. et al., 2021). Furthermore, high-altitude environments can considerably affect and homogenize the rumen microbial composition of cattle and sheep (Zhang et al., 2016). Therefore, the changes in the ruminal bacterial community in Sanhe heifers from the LA to HA groups likely occurred because of the geographic environment and hypoxic stress at different altitudes.

In this study, we also investigated the effect of altitude on the bacterial core OTUs in the rumen of Sanhe heifers. The results showed that four genera correlated with DMI, *Rikenellaceae_RC9_gut_group*, *unidentified_f_Bacteroidales_S24-7_group*, and *Prevotella_1*, which belong to the phylum Bacteroidetes, and *Ruminiclostridium_6*, which belong to Firmicutes. In addition, altitude was negatively associated with the relative abundance of OTUs within the genera *Prevotellaceae_UCG-003*, *Prevotella_1*, and *Rikenellaceae_RC9_gut_group*, which belong to the phylum Bacteroidetes, and *Lachnospiraceae_XPB1014_group*, *Oribacterium*, *Eubacterium_cellulosolvens_group*, and *Olsenella*, which belonged to Firmicutes. Altitude was positively associated with the relative abundance of OTUs within *Rikenellaceae_RC9_gut_group*, *unidentified_f_Bacteroidales_S24-7_group*, and *Ruminiclostridium_6*, which belong to the phylum Bacteroidetes, and *Ruminococcaceae_UCG-010*, *Ruminococcaceae_NK4A214_group*, and *Succiniclasticum*, which belong to Firmicutes. Therefore, we speculate that these genera are essential for the adaptation of the bacterial community to high altitudes. Previous studies have shown that genera affected by altitude are involved in rumen production (Lin et al., 2019), immunity, and methane production (Pragna et al., 2018). In addition, Tsao et al. (2021) reported that fecal bacteria might be associated with the activity of antioxidant enzymes. Similarly, our results indicate that antioxidant enzyme activities and oxidant products such as SOD, GSH-Px, and H_2_O_2_ are likely associated with the genus *Prevotella_1* belonging to the family Prevotellaceae; *Olsenella* belonging to Coriobacteriaceae; *Ruminococcaceae_UCG-01*, *Ruminococcaceae_UCG-010*, and *Ruminococcaceae_NK4A214_group* belonging to Ruminococcaceae; and *Eubacterium_cellulosolvens_group* belonging to Lachnospiraceae. This suggests that rumen bacteria might affect the antioxidant capacity of Sanhe heifers at different altitudes. Similarly, the core OTUs presented a strong interaction correlation.

Gastrointestinal microorganisms are crucial for host–microbiome interactions, immune development, absorption and degradation of nutrients, and enzyme metabolism (Martin et al., 2010). According to the PICRUSt2 results, the bacterial composition of the rumen differed between the regions in this study, whereas the functional gene composition of rumen microbes was similar. Microbial communities often show remarkable taxonomic diversity. However, their functional compositions are not directly related to taxonomic diversity because similar gene functions might be exhibited by several concomitants but taxonomically distinct microbes (Louca et al., 2018).

## Conclusion

In this study, we analyzed digestion performance, rumen bacteria, and fermentation parameters in Sanhe heifers at different altitudes in China. The results indicated that the digestion performance and rumen bacteria changed according to the altitude. Therefore, we conclude that Sanhe cattle live in high-altitude environments by adjusting their rumen bacteria and digestion performance. This study provides insights into the adaptation mechanisms of Sanhe heifers to high altitudes. Future studies should further investigate these mechanisms.

## Data Availability Statement

The datasets presented in this study can be found in online repositories. The names of the repository/repositories and accession number(s) can be found in the article/Supplementary Material.

## Ethics Statement

The animal study was reviewed and approved by the Ethical Committee of the College of Animal Science and Technology of China Agricultural University. Written informed consent was obtained from the owners for the participation of their animals in this study.

## Author Contributions

XZ: conceptualization, methodology, investigation, and writing—original draft. SH: writing—review and editing. WW: reviewed and provided guidance for the manuscript. SL: project administration and supervision. All authors contributed to the article and approved the submitted version.

## Funding

The services used in this study were purchased by the Ministry of Agriculture and Rural Affairs: Experiment and Demonstration of Adaptive Production Technology for Dairy Cows in High Altitude Regions (no. 16190319).

## Conflict of Interest

The authors declare that the research was conducted in the absence of any commercial or financial relationships that could be construed as a potential conflict of interest.

## Publisher’s Note

All claims expressed in this article are solely those of the authors and do not necessarily represent those of their affiliated organizations, or those of the publisher, the editors and the reviewers. Any product that may be evaluated in this article, or claim that may be made by its manufacturer, is not guaranteed or endorsed by the publisher.

## Footnotes

^1^ https://github.com/picrust/picrust2
