# Supplementary material for: Effects of Altitude on the Digestion Performance, Serum Antioxidative Characteristics, Rumen Fermentation Parameters, and Rumen Bacteria of Sanhe Heifers
Source: Front Microbiol. 2022 Apr 28;13:875323. doi: 10.3389/fmicb.2022.875323 (PMC9097872; doi:10.3389/fmicb.2022.875323)
Supplement: Supplementary file 1 [file Data_Sheet_1.docx]

**Table S1. The ingredients and nutrient levels of the TMR (DM, %).**

| Items  Items | LA  Control | HA  HA |
| --- | --- | --- |
| Wheat bran | 12.07 | --- |
| Highland barley bran | --- | 10.59 |
| Corn silage | 29.69 | 29.59 |
| Wheat straw | 10.94 | --- |
| Leymus chinensis hay | 7.81 | --- |
| Oat hay | --- | 18.65 |
| Alfafa hay | --- | 7.84 |
| Whole cottonseed | 3.12 | 2.98 |
| Steam-flaked corn | --- | 15.14 |
| Cottonseed meal | 4.79 | 6.40 |
| Soybean meal | --- | 5.86 |
| Barley | 15.03 | --- |
| Rapeseed meal | 13.60 | --- |
| Sodium bicarbonate | 0.20 | 0.20 |
| Slow-release urea supplement | 0.30 | 0.30 |
| Dicalcium phosphate | 0.30 | 0.30 |
| Limestone | 0.60 | 0.60 |
| Salt | 0.30 | 0.30 |
| Premix^1^ | 1.25 | 1.25 |
| Total | 100.00 | 100.00 |
| Nutritional levels^2^ | | |
| Dry matter (DM) | 50.79 | 51.30 |
| Organic matter (OM) | 90.45 | 91.05 |
| Crude protein (CP) | 15.70 | 15.78 |
| Ether extract (EE) | 2.92 | 2.91 |
| Neutral detergent (NDF) fiber (NDF) | 38.98 | 38.85 |
| Acid detergent fiber (ADF) | 22.36 | 22.79 |
| Ca | 0.78 | 0.83 |
| P | 0.44 | 0.46 |
| NE_M_,^3^ MJ /kg | 6.70 | 6.82 |
| NE_G_,^4^ MJ/kg | 4.61 | 4.74 |

Note:^1^Premix provided the following per kg for the diets of Sanhe heifers: vitamin A, 265,000IU; vitamin D_3_, 110,200 IU; vitamin E, 23,000 IU; Mn, 0.25%; Fe, 0.25%; Zn, 0.25%; Cu, 15 mg; I, 265,000 IU; Se, 30 mg.

^2^Nutrient composition: They were actual measured values.

^3^NE_M_, [net energy for maintainance](http://m.shortof.com/suolueci/nem-net-energy-maintainance), was calculated according to NRC (2001).

^4^NE_G_, net energy for growth, was calculated according to NRC (2001).

**Table S2. The genera of relative abundance > 0.01% within the rumen bacteria of LA and HA as determined by the Wilcoxon test (Only shown the *P*<0.05).**

| Genera | | Groups1 | | | | SEM | *P*-value | |
| --- | --- | --- | --- | --- | --- | --- | --- | --- |
|  |  | LA | | HA | |  |  |  |
| Ruminococcaceae_UCG-010 | 0.545 | | 0.895 | | 0.075 | | | 0.034 |
| Treponema_2 | 0.300 | | 1.003 | | 0.150 | | | 0.017 |
| Pseudobutyrivibrio | 0.761 | | 0.445 | | 0.069 | | | 0.028 |
| Ruminococcus_1 | 0.770 | | 0.342 | | 0.083 | | | 0.009 |
| Olsenella | 0.785 | | 0.282 | | 0.105 | | | 0.015 |
| U29-B03 | 0.056 | | 0.297 | | 0.085 | | | 0.026 |
| Ruminococcaceae_UCG-002 | 0.086 | | 0.197 | | 0.025 | | | 0.045 |
| Ruminiclostridium_9 | 0.069 | | 0.152 | | 0.029 | | | 0.034 |
| Eubacterium_brachy_group | 0.043 | | 0.165 | | 0.025 | | | 0.006 |
| Oribacterium | 0.135 | | 0.053 | | 0.017 | | | 0.015 |
| Lachnospira | 0.175 | | 0.011 | | 0.028 | | | <0.001 |
| unidentified_rumen_bacterium_RFN41 | 0.047 | | 0.099 | | 0.012 | | | 0.034 |
| Ruminiclostridium_6 | 0.037 | | 0.101 | | 0.012 | | | 0.002 |
| Lachnospiraceae_UCG-008 | 0.040 | | 0.097 | | 0.013 | | | 0.049 |
| Megasphaera | 0.000 | | 0.122 | | 0.036 | | | 0.001 |
| Lachnospiraceae_NK4A136_group | 0.091 | | 0.024 | | 0.012 | | | 0.003 |
| Lachnoclostridium_10 | 0.077 | | 0.029 | | 0.012 | | | 0.041 |
| Selenomonas | 0.000 | | 0.087 | | 0.023 | | | 0.002 |
| Erysipelotrichaceae_UCG-009 | 0.050 | | 0.019 | | 0.006 | | | 0.010 |
| Eubacterium_cellulosolvens_group | 0.048 | | 0.010 | | 0.006 | | | 0.001 |
| Prevotella_7 | 0.010 | | 0.044 | | 0.012 | | | 0.021 |
| Streptococcus | 0.013 | | 0.040 | | 0.006 | | | 0.006 |
| Corynebacterium_1 | 0.049 | | 0.003 | | 0.012 | | | 0.005 |
| Howardella | 0.043 | | 0.002 | | 0.008 | | | <0.001 |
| Elusimicrobium | 0.007 | | 0.034 | | 0.009 | | | 0.014 |
| Solanum_torvum | 0.001 | | 0.030 | | 0.005 | | | <0.001 |
| Lactobacillus | 0.002 | | 0.027 | | 0.005 | | | <0.001 |
| Lachnobacterium | 0.006 | | 0.022 | | 0.003 | | | 0.014 |
| Pyramidobacter | 0.001 | | 0.021 | | 0.004 | | | <0.001 |

^1^LA represents the low-altitude region (Hulunbuir City, Inner Mongolia Autonomous Region, 119°57 'E, 47°17' N; about 700 m altitudes, LA); HA represents the high-altitude region (Lhasa City, Tibet Autonomous Region 91°06'E, 29°36'N; about 3 750 m altitudes, HA).

**Table S3 Functional predictions for rumen bacteria with significantly different KEGG pathways for the three elevations (Only pathways level III which is significantly different and the abundances >0.01% are shown).**

| KEGG_Pathways | Groups | | SEM | *P*-value |
| --- | --- | --- | --- | --- |
| Level I/ Level III | LA | HA |  |  |
| Metabolism | 79.49 | 79.24 | 0.144 | 0.315 |
| Selenocompound metabolism | 0.99 | 1.07 | 0.011 | <0.001 |
| Biosynthesis of vancomycin group antibiotics | 2.46 | 2.56 | 0.016 | 0.002 |
| Taurine and hypotaurine metabolism | 0.63 | 0.66 | 0.005 | <0.001 |
| Geraniol degradation | 0.08 | 0.12 | 0.007 | <0.001 |
| Carotenoid biosynthesis | 0.06 | 0.01 | 0.001 | 0.003 |
| Thiamine metabolism | 1.75 | 1.63 | 0.022 | 0.001 |
| Valine, leucine and isoleucine degradation | 0.44 | 0.52 | 0.012 | <0.001 |
| Sulfur metabolism | 0.74 | 0.76 | 0.004 | 0.002 |
| Fatty acid degradation | 0.32 | 0.35 | 0.005 | <0.001 |

^1^LA represents the low-altitude region (Hulunbuir City, Inner Mongolia Autonomous Region, 119°57 'E, 47°17' N; about 700 m altitudes, LA); HA represents the high-altitude region (Lhasa City, Tibet Autonomous Region 91°06'E, 29°36'N; about 3 750 m altitudes, HA).

# Fig. S1 | Individual OTU rarefaction curves for each Sanhe heifer rumen sample take.


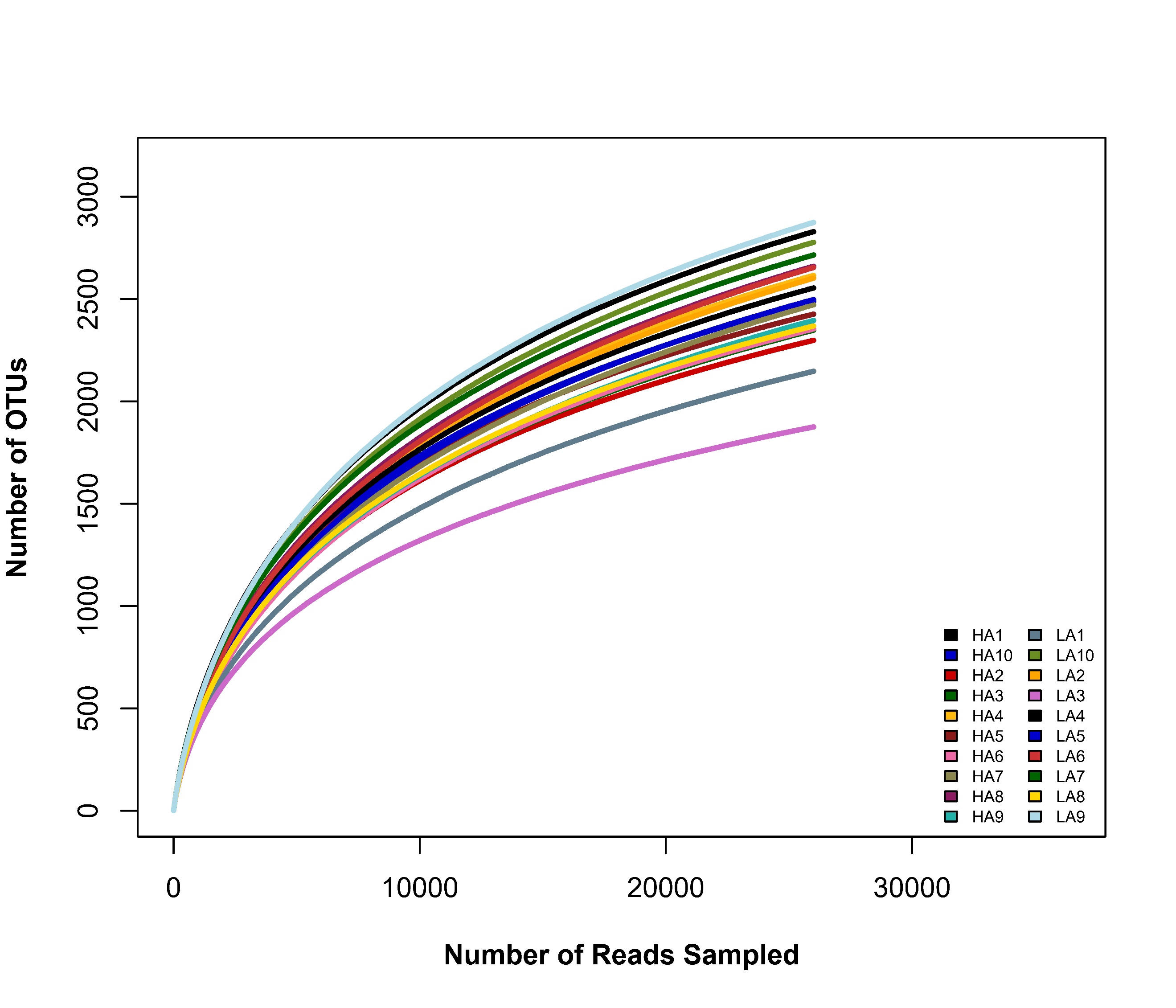


^1^LA represents the low-altitude region (Hulunbuir City, Inner Mongolia Autonomous Region, 119°57 'E, 47°17' N; about 700 m altitudes, LA); HA represents the high-altitude region (Lhasa City, Tibet Autonomous Region 91°06'E, 29°36'N; about 3 750 m altitudes, HA).

# Fig. S2 | Violin plots of number of OTUs, Chao1 richness and Shannon diversity index between LA and HA.


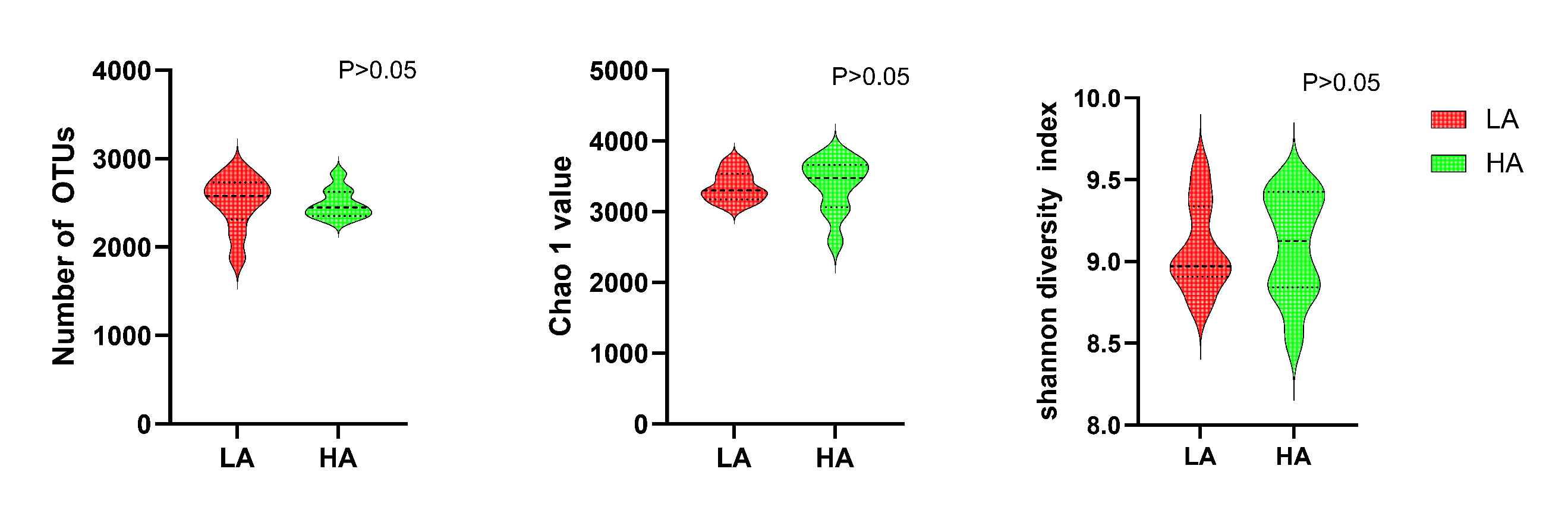


^1^LA represents the low-altitude region (Hulunbuir City, Inner Mongolia Autonomous Region, 119°57 'E, 47°17' N; about 700 m altitudes, LA); HA represents the high-altitude region (Lhasa City, Tibet Autonomous Region 91°06'E, 29°36'N; about 3 750 m altitudes, HA).
